# MRI-Guided Multi-Catheter High-Dose-Rate Interstitial Brachytherapy for Uterine Cervical Cancer

**DOI:** 10.3390/cancers17050770

**Published:** 2025-02-24

**Authors:** Hideya Yamazaki, Koji Masui, Ken Yoshida, Gen Suzuki, Tadashi Takenaka, Kei Yamada, Tadayuki Kotsuma, Yuji Takaoka, Kei Fujiwara, Yutaka Tanaka, Eiichi Tanaka

**Affiliations:** 1 Department of Radiology, Graduate School of Medical Science, Kyoto Prefectural University of Medicine, 465 Kajiicho Kawaramachi Hirokoji, Kamigyo-ku, Kyoto 602-8566, Japan; mc0515kj@koto.kpu-m.ac.jp (K.M.); gensuzu@koto.kpu-m.ac.jp (G.S.); ttakenak@koto.kpu-m.ac.jp (T.T.); kyamada@koto.kpu-m.ac.jp (K.Y.); 2 Department of Radiation Oncology, National Hospital Organization Osaka National Hospital, Osaka 540-0006, Japan; yoshidaisbt@gmail.com (K.Y.); t-kotsuma@osakah.johas.go.jp (T.K.); takaoka@saito-yukoukai-hp.jp (Y.T.); kh2102tt@gmail.com (K.F.); tanaka.yutaka.py@mail.hosp.go.jp (Y.T.); tanaka.eiichi.er@mail.hosp.go.jp (E.T.); 3 Department of Radiology, Saito Yukoukai Hospital, Ibaraki 567-0085, Japan

**Keywords:** cervical cancer, MRI-guided brachytherapy, interstitial brachytherapy

## Abstract

**Background/Objectives** To evaluate the efficacy and safety of unique magnetic resonance imaging (MRI)-guided interstitial brachytherapy (ISBT) for locally advanced cervical cancer that is unsuitable for intracavitary brachytherapy (ICBT) or intracavitary/interstitial brachytherapy (ICISBT). **Methods**: We analyzed the clinical outcomes, including toxicity, of 68 previously untreated patients with cervical cancer treated between 2014 and 2024. **Results**: The median high-risk clinical target volume (HR-CTV) was 53.20 cc (range, 16.34–147.03 cc) with ISBT. With a median follow-up time of 37.5 months (7–115 months), the three-year local control, progression-free survival, and overall survival rates were 89.8%, 52.4%, and 70.9%, respectively. Multivariate analyses showed significant associations of histology with local control, overall treatment times, HR-CTV volume with overall survival rate, over all treatment times, and HR-CTV volume and M category with progression-free survival. Toxicity grade 3 occurred in 12 patients (17.6%) and consisted of four genitourinary (5.8%) and seven gastrointestinal (10.2%) toxicities. **Conclusions**: MRI-guided ISBT is an effective treatment strategy for obtaining a favorable local control rate for selected advanced diseases with an acceptable complication rate. Future research is warranted to elucidate who would be good candidates for MRI-guided ISBT.

## Simple Summary

Interstitial brachytherapy (ISBT) is able to achieve good dose distribution regardless of the shape and size of the tumor and the utility of the vaginal, uterine cavity. We explored the potential of MRI-guided multi-catheter high-dose-rate ISBT in patients with locally advanced cervical cancer that was unsuitable for intracavitary brachytherapy or intracavitary/interstitial brachytherapy and obtained a favorable local control rate with an acceptable complication rate.

## 1. Introduction

Cervical cancer is the most common gynecological malignancy worldwide, accounting for more than 340,000 deaths in 2020 [1]. The standard treatment includes concurrent chemoradiotherapy with brachytherapy [2]. MRI-guided brachytherapy has been shown to improve local and pelvic control in these patients [3]. European (GEC-ESTRO Working Group) brachytherapy groups and the United States (ABS Image-guided Brachytherapy Working Group) established guidelines and recommendations for MRI-based intracavitary brachytherapy (ICBT) [4]. Additional needle implantation is used in cases where image-based ICBT alone is insufficient for target coverage, such as in intracavitary/interstitial brachytherapy (ICISBT) [5].

Interstitial brachytherapy (ISBT) is an effective treatment modality for advanced tumors, as treatment applicators can be implanted in and/or around the tumor without the limitations of tandem or ovoid applicators [6]. ISBT provides superior tumor coverage regardless of tumor size and extension and the utility of the vaginal and uterine cavity, and several studies have demonstrated the potential of ISBT with good local control results [7,8,9]. To utilize MRI images effectively, we designed a unique transrectal ultrasonography-guided plastic needle insertion ambulatory technique, combined with CT/MRI image-guided planning. This approach was validated through dose-volume histogram analysis and preliminary results from a single institution (Osaka National Hospital) [10]. The technique was later implemented at another institution (Kyoto Prefectural University of Medicine) [11], and the indication/eligibility criteria for ICBT, ICISBT, and ISBT using multi-institutional data were examined [12]. ISBT is utilized for advanced diseases where ICBT or ICISBT fails to achieve adequate tumor coverage. The purpose of the present study was to evaluate the efficacy and toxicity of MRI-guided ISBT for locally advanced cervical cancer that is unsuitable for intracavitary brachytherapy (ICBT) or intracavitary/interstitial brachytherapy (ICISBT) based on multi-institutional data.

## 2. Materials and Methods

### 2.1. Patient and Treatment

Between May 2014 and March 2024, 68 patients with uterine cervical cancer (median age, 56 years; range, 34–79 years) underwent ISBT at the Department of Radiology, National Hospital Organization, Osaka National Hospital, and Kyoto Prefectural University of Medicine (Table 1). The survivors were followed up for a minimum of 1 year, with a median follow-up period of 36 months (range, 7–110 months). The eligibility criteria for undergoing ISBT were determined based on ABS recommendations (bulky lesions, narrow vagina, inability to access the cervical os, extension to the lateral parametrium or pelvic sidewall, and lower vaginal extension, bladder, or rectal invasion) [13].

Histological findings showed 62 cases of squamous cell carcinomas and 6 cases of other histological types. According to the 7th UICC classification of 2010, the cohort included 9 T2 (2 a2 and 7 2b), 44 T3 (10 T3a and 34 T3b), and 15 T4 lesions. There were 19 N0 and 49 N1 patients, with 11 patients classified as having M1 para-aortic lymph node (PALN) metastases. All the patients received external beam radiotherapy (EBRT) to the entire pelvis, with a median prescribed dose of 50 Gy (range, 30–50.47). In addition, 34 patients underwent center-shielded (CS) EBRT (median, 10 Gy; range, 4–30 Gy). Additional boost irradiation for pelvic lymph node metastases was administered to 32 patients (median dose, 9 Gy; range, 2–10 Gy). EBRT for PALN was performed in 11 patients (median 55 Gy; 50.4–60 Gy). The median overall treatment time was 51 days (range, 31–82 days). We performed ISBT after whole-pelvic EBRT and before CS EBRT (Supplemental Figure S1). In principle, the midline block of CS EBRT is decided according to the treatment volume of the ISBT.

The details of ISBT have been described previously [10,11]. In brief, MRI and transrectal ultrasonography were used to differentiate between ICBT, ICBT/ICISBT, and ISBT. The decision to perform ISBT was made before the initiation of EBRT. ISBT required an operating room for the procedure. However, it was possible to change the adaptation of ISBT when EBRT was more effective than expected, and patients underwent ICBT or ICISBT. An ambulatory technique was adopted [10]. All the patients underwent CT-based planning with MRI reference images for contouring the high-risk clinical target volume (HR-CTV) and organs at risk (OARs), including the rectum, bladder, and sigmoid colon, as required. We used rigid registration for fusion of CT and MRI images taken in same position after applicator implantation.

These contours were defined based on the recommendations of the Gynecological GEC-ESTRO Working Group for reporting 3D-sectional image-assisted brachytherapy for cervical cancer [14,15]. The HR-CTV was delineated on axial T2-weighted MR images, with manual modifications after computer optimization. The single-fraction dose was 5–6 Gy, and the median total prescribed dose was 30 Gy/5 in the fractions (range, 24–30 Gy).

Fifty-seven patients (83.8%) received concurrent chemotherapy, including forty-two patients (61.7%) receiving cisplatin regimens (40 mg/m^2^, q1week). The other regimens included seven Paclitaxel + Carboplatin and three nedaplatin regimens. The remaining 11 patients did not receive chemotherapy due to their advanced age, poor performance status, or impaired organ function.

The equivalent dose in 2 Gy fractions (EQD2) was calculated by combining the EBRT and brachytherapy doses (D90), using α/β = 10 for tumors and α/β = 3 for OARs. The National Cancer Institute Common Terminology Criteria for Adverse Events (version 5.0) were used to classify the complications.

We analyzed overall survival (OS), progression-free survival (PFS), local control rate (LC), and toxicity as endpoints.

All the patients included in the analysis provided written informed consent. This study was conducted in accordance with the principles of the Declaration of Helsinki and approved by the Institutional Review Board of Kyoto Prefectural University (ERB-C-776).

### 2.2. Statistical Analyses

StatView 5.0 and the EZR stat package version 1.65 was used for statistical analysis [16]. Percentages were analyzed using Fisher’s exact test, and Student’s t-tests were performed for normally distributed data. The Mann–Whitney U-test for skewed data was performed for comparison. The Kaplan–Meier method was used to analyze the OS, PFS, and LC, all of which were calculated beginning from the first day of PBT. Multivariate Cox regression models were applied for OS, PFS, and LC. The candidate covariates in these models were age (continuous), overall treatment time (OTT) (<50 days vs. ≥50 days), EQD2 (<90 Gy vs. ≥90 Gy), HR-CTV (<60 cc vs. ≥60 cc), histology (squamous cell carcinoma C vs. others), T (2 vs. 3 vs. 4), lymph node (pelvic LN (+) vs. (−), M (para-aortic LN (+) vs. (−)), and concurrent chemotherapy (yes vs. no). Variable selection for multivariate models was conducted using the stepwise method with the AIC, and *p* < 0.05 was considered statistically significant. Cut-off values for HR-CTV, OTT, and EQD2 were calculated using receiver operating characteristic analysis or set at the median or mean value when not specified.

## 3. Results

The treatment parameters are listed in Table 1. The median volume of HR-CTV was 53.20 cc (range, 16.34–147.03 cc). The median D90 per fraction was 6.78 Gy (range, 5.03–8.03 Gy). When the EBRT dose was combined with the brachytherapy D90, the total EQD2 was 89.5 Gy in the EQD2 (range, 80.0–100.0 Gy). The detailed treatment parameters for each T category and stage are shown in Supplemental Tables S1 and S2. The D2cc (rectum) per fraction was 4.49 Gy (range, 3.10–6.57 Gy) and 77.45 Gy (range, 50.8–111.9 Gy) in the EQD2 for all treatments, and the D2cc (bladder) per fraction was 5.32 Gy (range, 3.13–8.13 Gy) and 86.05 Gy (range, 54.1–140.6 Gy) in the EQD2 for all treatments.

**Figure 1:**
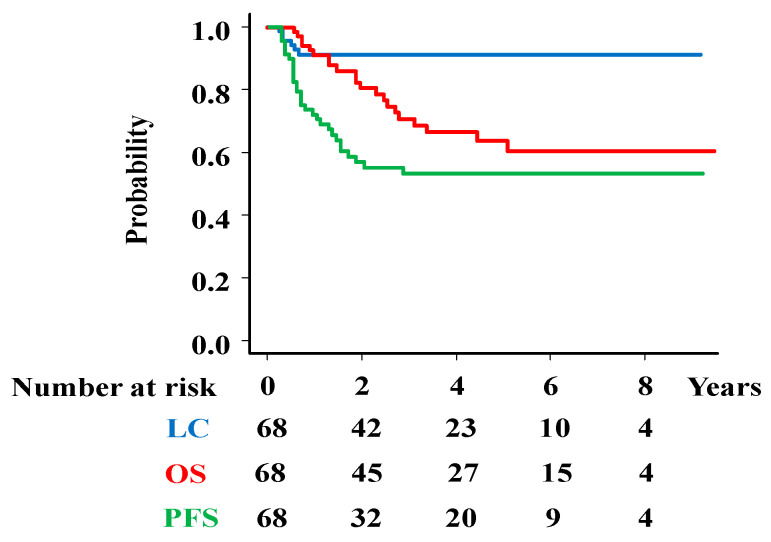
Survival analysis. Overall survival (OS), progression-free survival (PFS), and local control (LC) rates in total population.

The median follow-up time was 37.5 months (7–115 months) for the total population and 48 months (12–115 months) for the survivors. Local failure occurred in six patients (8.8%), and the 3-year LC rate was 91.2% (95%CI = 81.4–95.9%, Figure 1). The 3-year LC rates were 100%, 57.1%, 100%, 97.1%, and 86.7% for the T2a2, T2b, T3a, T3b, and T4 stages, respectively (Supplemental Table S1).

**Figure 2:**
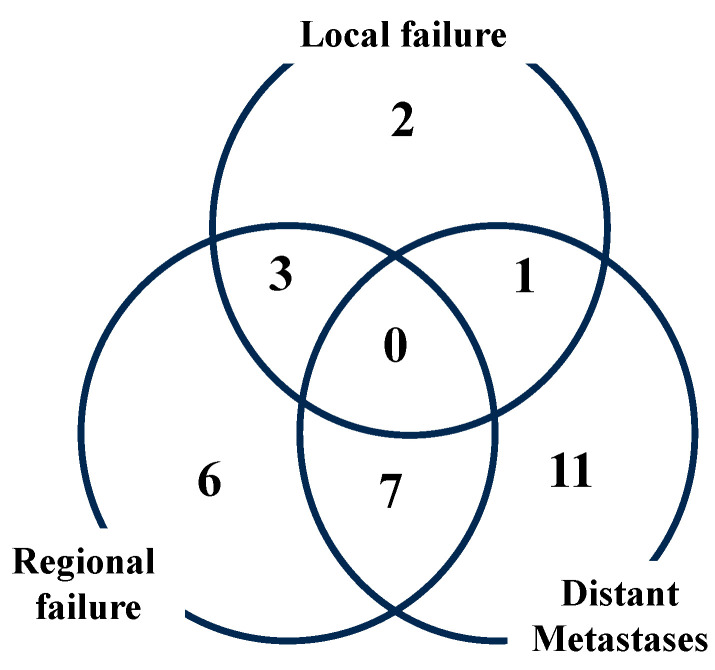
Failure pattern of recurrence.

The 3-year PFS was 52.4% (95%CI = 39.4–63.8%, Figure 1), including 16 pelvic lymph node recurrences (23.5%), 19 distant metastases (27.9%), 18 distant metastases and 1 para-aortic lymph node metastasis (Figure 2).

At the final follow-up, 47 patients were alive, while 21 patients had died. The causes of death included tumor progression (n = 17), other illnesses, or unknown reasons (n = 5). The 3-year overall survival rate was 70.9% (95%CI = 57.1–80.9%, Figure 1) [5-year OS rate; 63.9% (49.3–75.3%)]. The 3-year OS rates were 100%, 100%, 66.7%, 51.9%, 57.1%, and 36.4% for patients with stages 2A, 2 B, 3A, 3 B, 4A, and 4B disease, respectively (Supplemental Table S2).

Multivariate analyses (Table 2) were performed to evaluate factors associated with LC, OS, and PFS.

**Figure 3:**
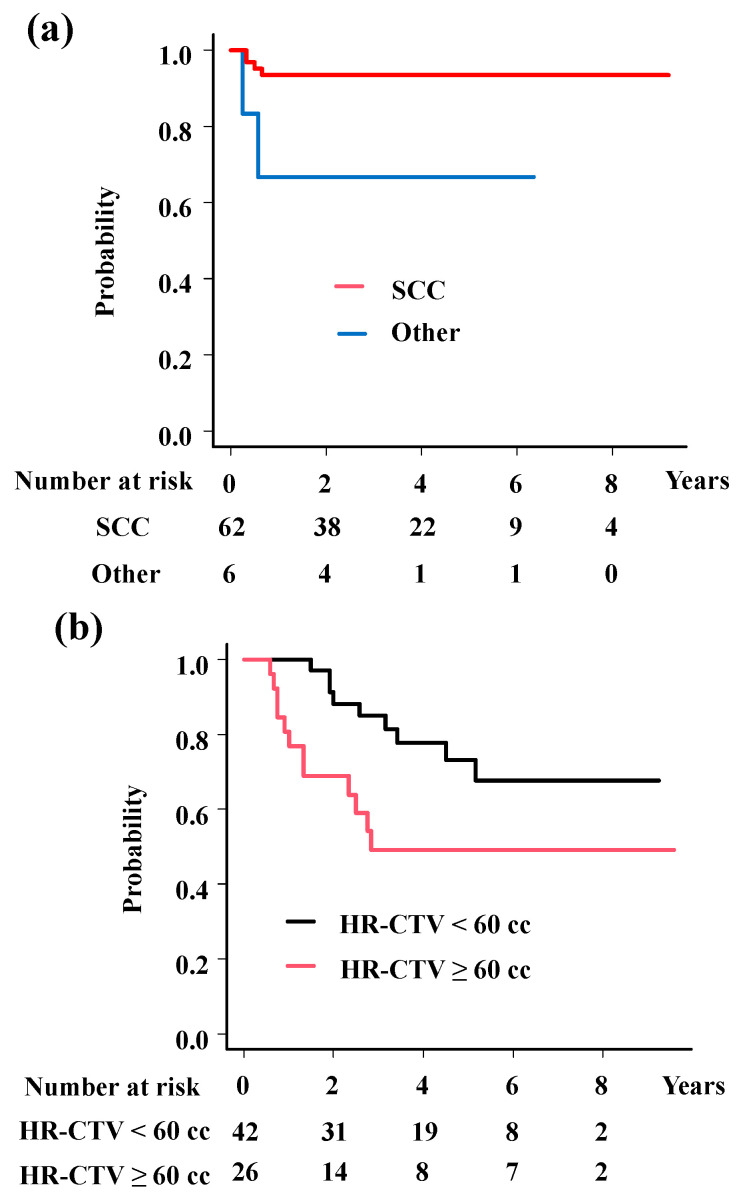

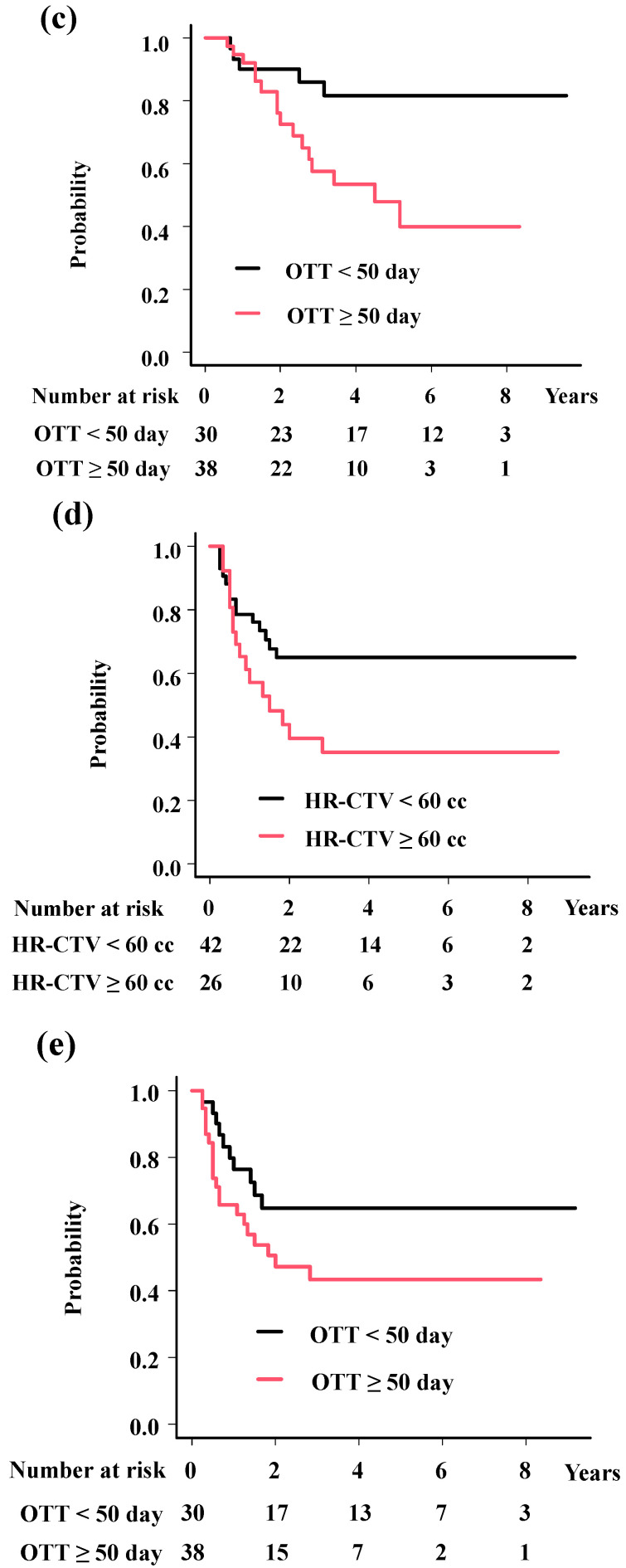

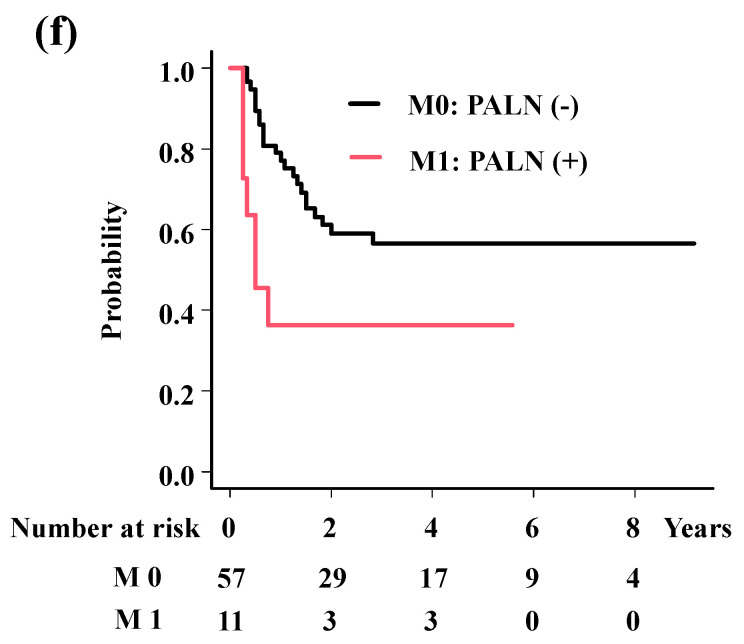
Survival analysis according to predisposing factors. (**a**) Local control rates according to histology. The 3-year local control rates were 93.5% and 66.7% for squamous cell carcinoma and others (*p* = 0.0173). (**b**) Overall survival rates according to volume of HR-CTV. The 3-year overall survival rates were 85.0% and 49.2% for patients with HR-CTV < 60 cc and HR-CTV ≥ 60 cc (*p* = 0.017). (**c**) Overall survival rates according to overall treatment time. The 3-year overall survival rates were 85.9% and 57.5% for patients with OTT < 50 days and OTT ≥ 50 days (*p* = 0.0123). (**d**) Progression-free survival time according to volume of HR-CTV. The 3-year progression-free survival rates were 64.9% and 35.1%, for patients with HR-CTV < 60 cc and HR-CTV ≥ 60 cc (*p* = 0.0386). (**e**) Progression-free survival time according to overall treatment time. The 3-year progression-free survival rates were 64.8% and 43.3% for patients with OTT < 50 days and OTT ≥ 50 days (*p* = 0.0875). (**f**) Progression-free survival time according to M category. The 3-year progression-free survival rates were 56.6% and 36.4% for patients with para-aortic lymph node (−) and (+) (*p* = 0.0171). *p* values were calculated with log-rank test.

The analyses showed significant associations of histology (HR = 0.16; 95%CI = 0.03–0.9, *p* = 0.037, Figure 3a) with LC; HR-CTV volume (HR = 5.76; 95%CI = 2.17–15.27, *p* = 0.00044, Figure 3b) and OTT (HR = 6.69; 95%CI = 2.21–20.26, *p* = 0.00078, Figure 3c) with OS; and HR-CTV volume (HR = 2.19; 95%CI = 1.05–4.57, *p* = 0.036, Figure 3d), OTT (HR = 2.21; 95%CI = 1.02–4.77, *p* = 0.045, Figure 3e), and M category (HR = 2.47; 95%CI = 1.04–5.85, *p* = 0.04, Figure 3f) with PFS.

Grade 3 toxicity occurred in 12 patients (17.6%), including 3 cases of acute toxicity and 11 cases of late toxicity. There were four genitourinary (5.8%) and seven gastrointestinal (10.2%) toxicities, with three cases of fistulae (4.4%, comprising rectovaginal, vesicovaginal, and small intestine–vaginal fistulae).

## 4. Discussion

**Figure 4:**
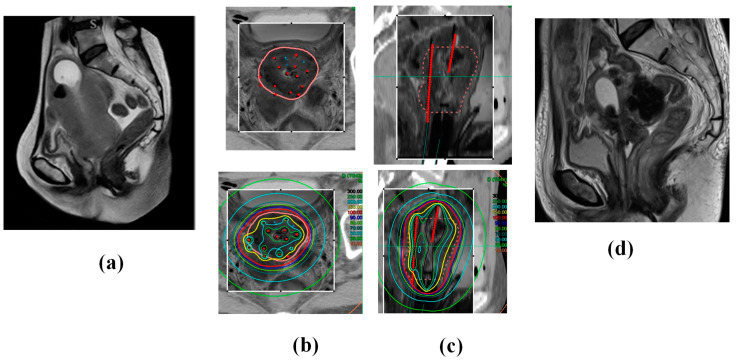
A 68-year-old woman presented with cervical cancer with uterine corpus invasion cT3bN1M0 (Stage 3B). The tumor exhibited significant extension into the lateral pelvic wall and corpus, with an initial gross tumor volume of 190.7 cc. The patient underwent whole-pelvic external beam radiotherapy (WP EBRT; 30 Gy in 15 fractions) with concurrent chemotherapy (40 mg/m^2^, q1 week CDDP × 5 times). The tumor volume reduced to 133.3 cc, and the patient underwent ISBT (30 Gy/5 fractions in 3 days administered twice daily). She continued to receive CS EBRT (10 Gy/10 fractions) and a lymph node boost EBRT (6 Gy/3 fractions). The patient remains in complete remission and alive 38 months post-treatment, with no evidence of recurrence. (**a**) Initial MRI image (Sagittal image). (**b**) Axial images and (**c**) Sagittal images of CT (outside of the rectangle) and MRI (inside of the rectangle) fusion images used in ISBT planning taken after the ISBT procedure. The pink line in the upper panel indicates the HR-CTV, and the red points and lines indicate the applicators. Note 100% isodose line (red line) fit to the HR-CTV in the bottom panel. (**d**) MRI image 4 months later (complete remission status).

Using multi-institutional data, we reported and confirmed the efficacy and toxicity of MRI-guided ISBT for locally advanced cervical cancer that is unsuitable for ICBT or ICISBT. This unique ISBT technique offers several merits, enabling higher tumor doses, even in cases of large, irregularly shaped tumors with extensive spread or difficulty inserting applicators (Figure 4; uterine corpus extension case).

In addition, this technique shortened the OTT compared to the conventional ‘once a week’ brachytherapy schedule (ICBT and IC/ ICISBT) in Japan (Supplemental Figure S1), requiring only one MRI session for treatment planning. To our knowledge, this study represents the first report of MRI-guided ISBT for untreated cervical cancer in a cohort exceeding 60 patients.

The Manchester treatment system was the gold standard (Point A dose prescription) in the 20th century and has changed to image-based ICBT (HR-CTV volume dose prescription) [17]. ABS and GEC-ESTRO published guidelines for image-guided brachytherapy and interstitial techniques for several conditions because of their better dose distribution, not only for tumor control but also for toxicity reduction in organs at risk [4,14,15]. Image-guided brachytherapy has become a global standard, and the EMRACE studies have shown excellent outcomes with interstitial techniques in ICISBT [18].

Based on these recommendations, we explored the indications for interstitial techniques [10]. First, HR-CTV volume is an important factor in determining the appropriate approach [18]. We hypothesized that a distance of <2 cm between the HR-CTV and applicator axis (HR-CTV diameter < 4 cm if the applicator is located in the central area) represents a threshold for the application of the interstitial technique, which was calculated as 33 cc (approximately 30 cc) in a sphere volume. Therefore, ICIS-BT or ISBT is a better choice for patients with an HR-CTV > 30 cc. Furthermore, if the distance between the HR-CTV and applicator tip increase to 2.5–3 cm (HR-CTV diameter < 5–6 cm, HR-CTV volume of 65–113 cc, approximately > 60 cc), we speculate that ISBT is a feasible choice for achieving adequate dose distribution. Takenaka et al. examined the real data of 112 patients treated with brachytherapy (54 ICBT, 11 ICISBT, 47 ISBT) in these institutions. They reported that the average GTV at diagnosis was 80.9 cc (5.4 cm in diameter, range, 4.4–343.2 cc), which reduced to 20.6 cc (3.5 cm in diameter, 25.5% of initial volume, range, 0.0–124.8 cc) at initial brachytherapy. As the initial tumor volume reduced to 25.5% (74.5% reduction) after external beam radiotherapy with concurrent chemotherapy [12], it could be supposed that an initial GTV volume of 116 cc (≈6 cm diameter) could be a good threshold for the requirement of an interstitial technique (which may be reduced to 30 cc at brachytherapy). In addition, patients with an initial GTV of 233 cc or more (which may be reduced to 60 cc during brachytherapy) could be candidates for ISBT. In real-world data, all patients with initial GTV > 150 cc underwent ISBT, and ISBT could deliver higher D90 (89.10 Gy range, 65.5–107.6 Gy) than those of ICISBT (73.94 Gy, range, 71.44–82.50 Gy) and ICBT (72.83 Gy, range, 62.50–82.27 Gy) (*p* < 0.0001) [12]. ABS and the Executive Summary of an American Society for Radiation Oncology Clinical Practice Guideline recommend achieving D90 ≥ 80 Gy for tumor control and D90 ≥ 85 Gy for patients with poor response or large volume (tumor diameter > 4 cm ≈ 33 cc) disease [15]. Our technique achieved the required dose for the tumor.

Schmid et al. reported (The EMBRACE-I study) that factors such as histology, D90 to HR-CT, maximum tumor dimension, CTVHR < 45 cc, OTT, tumor necrosis on MRI at diagnosis, uterine corpus infiltration at diagnosis on MR-IGABT, and mesorectal infiltration on MR-IGABT had a significant impact on LF [19]. Our findings align with these conclusions. In some cases, ovoid and tandem applicators pose challenges due to vertical extensions, such as uterine corpus involvement, which hinder adequate tandem positioning and dose distribution in ICISBT. This issue is frequently observed in patients with local disease relapse [14]. In this regard, ISBT is a good indication for achieving sufficient dose distribution, even when corpus invasion results in an irregular shape (Supplemental Figure S1).

OTT remains a critical prognostic factor, as established in the 20th century. We confirmed its significance in ISBT; Mazeron et al. reported a threshold of 56 days, and Potter et al. recommended OTT within 51 days in the EMBRACE study. Our data concurred with these recommendations [18,20]. Despite favorable LC rates, improving tumor control beyond the HR-CTV is essential for better PFS and OS. We speculate that there is a potential to reduce the OTT if we use the simultaneous integrated boost technique to boost EBRT for lymph node metastases. In addition, our findings indicated that M factor (para-aortic lymph node metastases) was an important predisposing factor for PFS. Advanced cases with para-aortic lymph node involvement tend to progress outside irradiated areas, underscoring the importance of systemic therapy. Exploring systemic agents, such as immune checkpoint inhibitors (ICI) may improve outcomes in locally advanced cervical cancer beyond the irradiated area [21].

This study acknowledges several limitations. First, the interstitial technique required an invasive procedure to insert applicators under anesthesia, which caused severe toxicity; although rare (i.e., bleeding during extraction of applicators, fistula in late phase), it should be performed in institutions with experienced multidisciplinary teams. Next, the CS technique used in Japan is unique and has several advantages in reducing toxicity [22,23,24]; however, it is not a standard technique used in Western countries [13,14,15]. CS (midline block) has been applied to lower the dose to the rectum and bladder and avoid severe complications because brachytherapy can provide a concentrated dose to the primary tumor. Although this practice has decreased over the years globally, the CS technique continues to be used as the standard technique in Japan [24]. The use of CS has resulted in a relatively low incidence of late complications in the rectum and bladder without compromising the disease control [24]. A recent study of composite dose distributions of the treatment regimen using central shielding revealed its characteristics, which explained the benefit of this technique [22,23,24]. The dose contributions of the CS were variable but not negligible. Tamaki et al. reported that the contributions of CS to the HR-CTV D90 values were 24–56% of the CS plan doses for a shielding width of 3 cm and were 13–35% for a shielding width of 4 cm [22]. Therefore, our estimated D90 values may be higher than the actual value.

Third, tumor response during chemoradiotherapy with EBRT is an important predisposing factor [25,26,27,28,29,30]. Ohtaka et al. reported that poorly responding tumors (reduction ratio < 68.8%) had poor prognosis in terms of OS, PFS, and LC [26]. Our findings concurred with their findings that the reduction rate could be an important factor in LC. Tumors with less than 65% reduction showed a poorer local tumor control rate than their counterparts (100% = 18/ 18 in the >65% reduction group vs. 72% = 8/11 in the <65% reduction rate group, *p* = 0.045). However, our study was limited by the initial tumor volume data, which were available for only half the patients. Despite these limitations, this comprehensive study highlights that ISBT is a viable treatment modality for cases unsuitable for ICBT or ICISBT. A prospective multicenter trial is required to define the clinical role of this treatment modality, and it is vital to use methodologies that can be easily replicated.

## 5. Conclusions

MRI-guided ISBT is an effective treatment strategy for achieving a favorable LC rate in selected advanced diseases with an acceptable complication rate. Future research is warranted to identify suitable candidates for MRI-guided ISBT.

## Figures and Tables

**Table 1:** Patients, tumor, and treatment characteristics.

Factor	Group	PTNO (%) or Median [Range]
		n = 68
Age		64.00 [28.00, 84.00]
Histology	Squamous cell carcinoma	62 (91.2)
	Other *	6 (8.8)
T category	2	9 (13.2)
	3	44 (64.7)
	4	15 (22.1)
N category	0	19 (27.9)
	1	49 (72.1)
M category (paraaortic lymph node)	0	57 (83.8)
	1	11 (16.2)
Stage classification	2A	1 (1.5)
	2B	1 (1.5)
	3A	6 (8.8)
	3B	35 (51.5)
	4A	14 (20.6)
	4B	11 (16.2)
Treatment		
EBRT (Dose)	(Gy)	56.00 [44.00, 64.00]
EBRT (fractions)		30.00 [20.00, 33.00]
Whole pelvis	(Gy)	50.00 [30.00, 50.47]
Central shield	(Gy)	4.00 [0.00, 30.00]
ISBT (dose/ fraction)	(Gy)	6.00 [5.00, 6.00]
ISBT total dose (dose)		25.00 [24.00, 30.00]
D90 of brachytherapy	(Gy)	6.78 [5.03, 8.03]
HR-CTV volume at brachytherapy	(cc)	53.20 [16.34, 147.03]
Total EQD2	(Gy)	89.5 [80–100]
Overall treatment time	(day)	51.00 [31.00, 82.00]
Concurrent chemotherapy	No	11 (16.2)
	Yes	57 (83.8)
	CDDP	42 (61.8)
	Other drug	15 (22.1)

Other * Adenocarcinoma 3, endometrioid carcinoma 1, adenosquamous carcinoma 1, mucinous adenocarcinoma 1. CDDP: cisplatin, EBRT: external beam radiotherapy, ISBT: interstitial brachytherapy, EQD2: equivalent dose in 2 Gy fractions, D90: minimal dose to 90% of high-risk clinical target volume (HR-CTV).

**Table 2:** Multivariate analysis of potential predictive factors for LC, OS, and PFS.

Endpoint	Variable	Category	PTNO	3-Year (%)	Hazard Ratio (95%CI)	*p*-Value
Local control	Histology	Squamous cell carcinoma	62	93.5	0.16 (0.03–0.9)	**0.037**
		Others	6	66.7		
Overall survival	HR-CTV volume	<60 cc	53	77.1	5.76 (2.17–15.27)	**0.00044**
		≥60 cc	15	49.5		
	OTT	<50 days	30	85.9	6.69 (2.21–20.26)	**0.00078**
		≥50 days	38	57.5		
Progression-free survival	HR-CTV volume	<60 cc	53	58	2.19 (1.05–4.57)	**0.036**
		≥60 cc	15	37.5		
	OTT	<50 days	30	64.8	2.21 (1.02–4.77)	**0.045**
		≥50 days	38	43.3		
	M category	Para-aortic LN (+)	11	36.4	2.47 (1.04–5.85)	**0.04**
		Para-aortic LN (−)	57	56.6		

Statistically significant values are depicted in bold letters, CI: confidence interval, PTNO: number of patients.

## Data Availability

The individual de-identified data will be available on reasonable request to the corresponding author.

## Supplementary Materials

The following supporting information can be downloaded at: https://www.mdpi.com/article/10.3390/cancers17050770/s1, Figure S1: Treatment scheme, Table S1: HR-CTV volume, dose, and outcomes according to T category, Table S2: HR-CTV volume, dose, and outcomes according to stage.

## Abbreviations

The following abbreviations are used in this manuscript:

MRI	Magnetic resonance imaging
ISBT	Interstitial brachytherapy
ICBT	Intracavitary brachytherapy
ICISBT	Intracavitary/interstitial brachytherapy
HR-CTV	High-risk clinical target volume
PALN	Para-aortic lymph node
CS	Center-shielded
EBRT	External beam radiotherapy
EQD2	The equivalent dose in 2 Gy fractions
OS	Overall survival
PFS	Progression-free survival
LC	Local control
OTT	Overall treatment time
