# Supplementary material for: MRI-Guided Multi-Catheter High-Dose-Rate Interstitial Brachytherapy for Uterine Cervical Cancer
Source: Cancers (Basel). 2025 Feb 24;17(5):770. doi: 10.3390/cancers17050770 (PMC11899140; doi:10.3390/cancers17050770)
Supplement: Supplementary file 1 [file cancers-17-00770-s001.zip › Figure S1.pptx]

## Slide 1
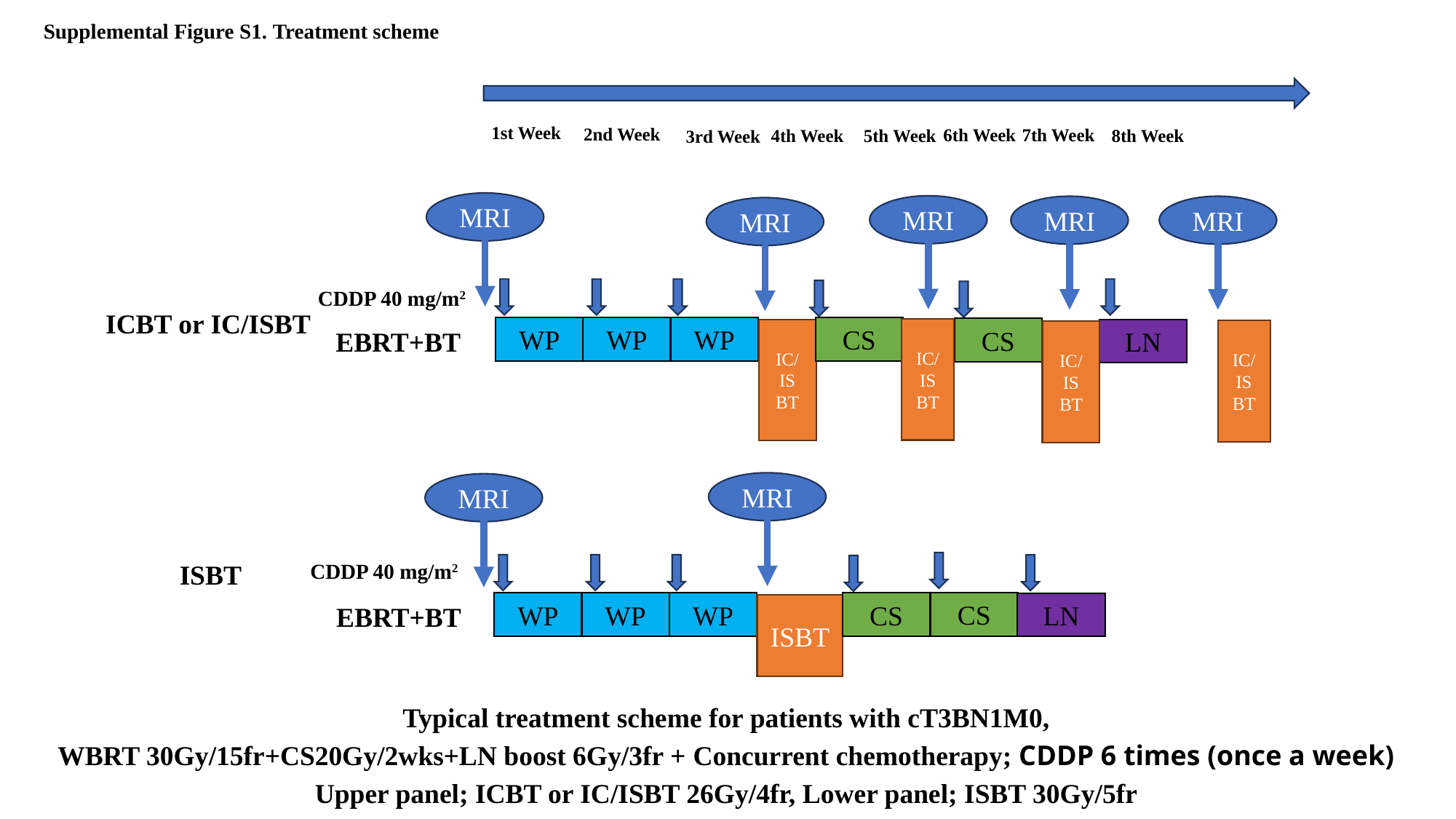

Supplemental Figure S1. Treatment scheme
1st Week
2nd Week
6th Week
7th Week
5th Week
8th Week
4th Week
3rd Week
MRI
MRI
MRI
MRI
MRI
CDDP 40 mg/m2
ICBT or IC/ISBT
IC/
IS
BT
EBRT+BT
WP
WP
WP
IC/
IS
BT
CS
CS
IC/
IS
BT
IC/
IS
BT
LN
MRI
MRI
ISBT
CDDP 40 mg/m2
CS
CS
EBRT+BT
WP
WP
WP
ISBT
LN
Typical treatment scheme for patients with cT3BN1M0,
WBRT 30Gy/15fr+CS20Gy/2wks+LN boost 6Gy/3fr + Concurrent chemotherapy; CDDP 6 times (once a week)
Upper panel; ICBT or IC/ISBT 26Gy/4fr, Lower panel; ISBT 30Gy/5fr
